# PCVMZM: Using the Probabilistic Classification Vector Machines Model Combined with a Zernike Moments Descriptor to Predict Protein–Protein Interactions from Protein Sequences

**DOI:** 10.3390/ijms18051029

**Published:** 2017-05-11

**Authors:** Yanbin Wang, Zhuhong You, Xiao Li, Xing Chen, Tonghai Jiang, Jingting Zhang

**Affiliations:** 1 Xinjiang Technical Institutes of Physics and Chemistry, Chinese Academy of Science, Urumqi 830011, China; wangyanbin15@mails.ucas.ac.cn (Y.W.); jth@ms.xjb.ac.cn (T.J.); 2 School of Information and Control Engineering, China University of Mining and Technology, Xuzhou 221116, China; xingchen@amss.ac.cn; 3 Department of Mathematics and Statistics, Henan University, Kaifeng 100190, China; zhangjingting15@mails.ucas.ac.cn

**Keywords:** proteins, position-specific scoring matrix, probabilistic classification vector machines

## Abstract

Protein–protein interactions (PPIs) are essential for most living organisms’ process. Thus, detecting PPIs is extremely important to understand the molecular mechanisms of biological systems. Although many PPIs data have been generated by high-throughput technologies for a variety of organisms, the whole interatom is still far from complete. In addition, the high-throughput technologies for detecting PPIs has some unavoidable defects, including time consumption, high cost, and high error rate. In recent years, with the development of machine learning, computational methods have been broadly used to predict PPIs, and can achieve good prediction rate. In this paper, we present here PCVMZM, a computational method based on a Probabilistic Classification Vector Machines (PCVM) model and Zernike moments (ZM) descriptor for predicting the PPIs from protein amino acids sequences. Specifically, a Zernike moments (ZM) descriptor is used to extract protein evolutionary information from Position-Specific Scoring Matrix (PSSM) generated by Position-Specific Iterated Basic Local Alignment Search Tool (PSI-BLAST). Then, PCVM classifier is used to infer the interactions among protein. When performed on PPIs datasets of *Yeast* and *H. Pylori*, the proposed method can achieve the average prediction accuracy of 94.48% and 91.25%, respectively. In order to further evaluate the performance of the proposed method, the state-of-the-art support vector machines (SVM) classifier is used and compares with the PCVM model. Experimental results on the *Yeast* dataset show that the performance of PCVM classifier is better than that of SVM classifier. The experimental results indicate that our proposed method is robust, powerful and feasible, which can be used as a helpful tool for proteomics research.

## 1. Introduction

Recognition of protein–protein interactions (PPIs) is essential for elucidating the function of proteins and further understanding the various biological processes in cells. In the last decade, a variety of biological methods have been used for large-scale PPIs detection, such as tandem affinity purification [1], yeast two-hybrid systems [2,3], and protein chip [4]. For the limit of the experimental technique, these methods have some disadvantages, including high cost and time-intensive, as well as high rates of both false-positive and false-negative. Hence, computational methods for the detection of protein interactions have become hot research topics of proteomics research. So far, a number of computational methods have been presented for the detection of PPIs based on different data types, such as protein domains, protein structure information, genomic information and phylogenetic profiles [5,6,7,8,9,10,11,12,13]. However, these approaches cannot be achieved unless prior information of the protein is available. Hence, the mentioned methods are not widespread. Compared to the rapid growth of a large number of protein sequences, other data that can be used to predict the PPIs are scarce. Therefore, computational methods using only protein amino acid sequence information for PPIs prediction is especially interesting [14]. Bock and Gough used a support vector machine (SVM) with protein sequence descriptors to predict PPIs [15]. Martin et al. proposed an approach to predict PPIs by using signature product, which is a descriptor that extends from signature descriptors [16]. Najafabadi et al. attempted to solve this problem with Bayesian network [17]. Shen et al. adopted a SVM model to predict PPI network by combining Skernel function of protein pairs with a conjoint triad feature [18]. Yu-An Huang et al. developed a method by combining discrete cosine transform and using weighted sparse representation-based classifier to predict PPIs, and it has achieved very exciting prediction accuracy when applying this method to detecting yeast PPIs [19]. Yan-Zhi Guo et al. also obtained promising prediction results by adopting support vector machine and auto covariance [20]. Loris Nanni et al. developed several matrix-based protein representation methods, including [21,22,23,24,25]. Other feature extraction approaches based on protein sequence have been proposed in [26,27,28,29,30,31,32,33,34]. In this study, a novel computational approach for predicting PPIs from amino acid sequences based on a probabilistic classification vector machines model (PCVM) and a Zernike moments descriptor (PCVMZM) was proposed. The major improvement is the development of a more accurate protein sequence representation. Specifically, we employed the Zernike moments feature representation on a Position-Specific Scoring Matrix (PSSM) to extract the evolutionary information from protein sequence, and then a probabilistic classification vector machines classifier is used to infer the PPIs. In more detail, a PSSM representation is used to represent each protein. Afterward, for the sake of obtaining more representative information, we apply a Zernike moments descriptor to extract features in each protein PSSM and use Zernike moments of 12-order information and generate a 42-dimensional feature vector. Finally, we adopt the machine learning method called PCVM to accomplish classification. The proposed method was applied to *Yeast* and *H. Pylori* PPIs datasets. The experiments have shown that a PCVM prediction model with a Zernike moments descriptor yields fantastic performance. By further contrast experiment, we found that our proposed method was superior to the state-of-the-art SVM, which clearly shows that the proposed approach is trustworthy in predicting PPIs [35,36,37,38,39].

## 2. Results and Discussion

### 2.1. Evaluation Measure

The proposed method is evaluated against the following criteria: The Accuracy (Acc), Sensitivity (Sen), Precision (Pre), and Matthew`s correlation coefficient (MCC). All the computational formula is defined as follows:

(1) $$\mathrm{Accuracy}=\frac{TP+TN}{TP+FP+TN+FN}$$

(2) $$\mathrm{Sensitivity}=\frac{TP}{TP+FN}$$

(3) $$\mathrm{Precision}=\frac{TP}{TP+FP}$$

(4) $$\mathrm{MCC}=\frac{\left(TP\times TN\right)-\left(FP\times FN\right)}{\sqrt{\left(TP+FN\right)\times \left(TN+FP\right)\times \left(TP+FP\right)\times \left(TN+FN\right)}}$$

where TP represents the number of true positive, that true samples are predicted correctly, TN represents the number of true negative that true noninteracting pairs are predicted correctly. FP represents the number of false positive that non-interacting pairs are predicted to be interaction. FN represents the number of false negative that interacting pairs are predicted to be non-interacting. In addition, the receiver operating characteristic (ROC) curve [40] is applied to evaluate the performance of our method. The area under an ROC curve (AUC) [41] also is computed.

### 2.2. Assessment of Prediction

In order to make our method more reliable, five-fold cross-validation was adopted to divide a whole dataset into five parts. Hence, we obtained five models through separate experiments for each data set. The prediction result of PCVM prediction models with a Zernike moments description of protein sequence on *Yeast* and *H. Pylori* datasets are shown in Table 1 and Table 2. From Table 1, we can see that our proposed method achieved a good performance on the *Yeast* dataset. Its average accuracy, sensitivity, precision, and MCC are 94.48%, 95.13%, 93.92% and 89.58%, respectively. When using our proposed method on the *H. Pylori* dataset, as shown in Table 2, we also achieved some satisfactory results of average accuracy, sensitivity, precision, and MCC of 91.25%, 92.05%, 90.60% and 84.04%, respectively.

From the experimental results, it can be seen that our proposed approach is robust, accurate and practical for predicting PPIs. The outstanding performance for detecting PPIs can be put down to the feature extraction and the classification model of our proposed method. It is effective that Zernike moments are used for feature extraction, and the PCVM model is accurate and robust in dealing with classification problems.

### 2.3. Comparison with the Support Vector Machine (SVM)-Based Method

In order to further evaluate the prediction performance of the proposed entire model, the SVM model is adopted based on the *Yeast* dataset to predict PPIs using the same Zernike moments to extract feature, and then, we compared the classification result between PCVM and SVM. We employed the SVM through the library for Support Vector Machines (LIBSVM) tool [42]. SVM have two parameters, *c* and *g*, respectively. A grid search method is used to optimize parameters *c* and *g*. In our experiment, a radial basis function is used as the kernel function and the initial value *c* and *g* was set to 0.4 and 0.5.

**Figure 1:**
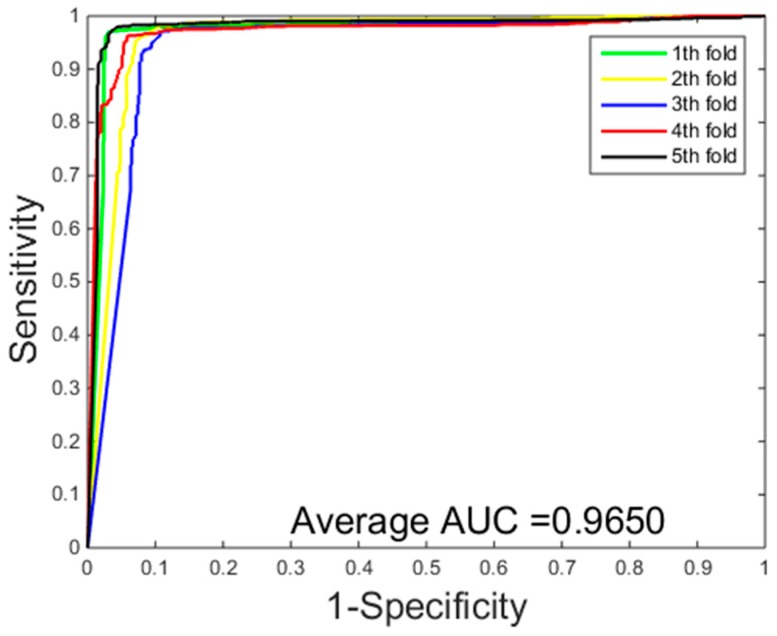
Receiver operating characteristic (ROC) curves performed of a probabilistic classification vector machines model (PCVM) on the *Yeast* dataset.

**Figure 2:**
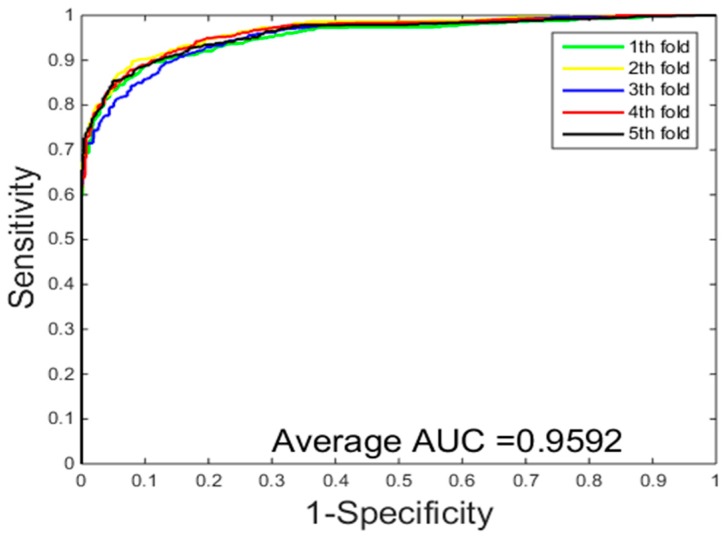
ROC curves performed of support vector machine (SVM) on the *Yeast* dataset.

Table 3 gives the prediction results of five-fold cross-validation over two different classification methods on the *Yeast* dataset. From Table 3, we can see that the classification method of SVM achieved 89.31% average accuracy, 87.54% average sensitivity, 90.81% average precision, 80.91% average MCC. While the classification results of the PCVM method achieved 94.48% average accuracy, 95.13% average sensitivity, 93.92% average precision, 89.58% average MCC. Experimental results show that PCVM classification method is significantly better than the SVM classification method. Comparison of ROC curves performed between RVM and SVM on the *Yeast* dataset from Figure 1 and Figure 2, we have experimental data obtained that the PCVM classifier is more accurate and robust than the SVM classifier for detecting PPIs.

The main improvement is attributed to three points: (1) the main advantage of PCVM is that the truncated Gaussian priors are adopted to generate robust and sparse results—in other words, the number of weight vectors is less than SVM. Hence, the complexity of the model is reduced, besides, the model is more general; (2) The parameter optimization procedure of the PCVM based on EM algorithm and probabilistic inference not only can improve the performance, but also save the effort to do cross-validation; (3) The PCVM model is simpler and easier to be understood, because the number of basic functions does not grow linearly with the number of training points. In general, the PCVM is a sparse model that makes up the shortcoming of SVM without deskilling the generalization performance and provides probabilistic outputs. Here it is, our proposed approach can produce satisfactory results.

### 2.4. Comparison with Other Methods

In recent years, many classification methods have been developed to predict PPIs. To further validate the performance of our proposed method, we compared the predictive performance of our method with other existing several well-known methods. The achieved results of five-fold cross-validation of different methods on the *Yeast* dataset and *H. pylori* dataset are shown in Table 4 and Table 5. From Table 4, the prediction accuracy of other previous methods on the *Yeast* dataset varies from 75.08% to 93.92%, while the proposed method achieved higher value of 94.48%. Similarly, the sensitivity and MCC of our method are also higher than those of other methods. We can find similar results on the *H. pylori* dataset in Table 5. Our proposed method achieves 91.25% accuracy, which is higher than the other five methods with the highest prediction accuracy of 87.50%. The same is true for precision, sensitivity and MCC. All prediction results in Table 4 and Table 5 indicate that the PCVM classifier is stable and robust and can improve the prediction performance compared with the state-of-the-art methods. The improvement of prediction performance of our method may derive from the novel feature extraction method which extracts the highly discriminative information, and the use of PCVM classifier which ensures accurate and stable prediction.

## 3. Materials and Methodology

### 3.1. Dataset

Up to now, many databases of PPIs data have been generated, such as Database of Interaction Proteins (DIP) [43], Molecular Interaction Database (MINT) [44], and Biomolecular Interaction Network Database (BIND) [45]. To evaluate our approach, we used two publicly available datasets: *Yeast* and *H. Pylori*, which were extracted from Database of Interaction Proteins (DIP). In order to ensure the reliability of the tests, we extract 5594 positive protein pairs to constitute the positive dataset and 5594 negative protein pairs to constitute the negative protein dataset from the *Yeast* dataset. Analogously, we extract 1458 positive protein pairs to constitute the positive dataset and 1458 negative protein pairs to constitute the negative protein dataset from the *H. Pylori* dataset. Therefore, the *Yeast* dataset consists of 11,188 protein pairs and the *H. Pylori* dataset consists of 2916 protein pairs.

### 3.2. Position-Specific Scoring Matrix

A Position-Specific Scoring Matrix (PSSM) was usually adopted to find distantly related proteins, protein disulfide, protein quaternary structural attributes and protein folding patterns [46,47,48,49]. In this paper, we also adopt PSSM to predict PPIs. Here, each protein was transformed into a PSSM matrix by employing the Position-Specific Iterated Basic Local Alignment Search Tool (PSI-BLAST) [50,51]. A PSSM is represented as

(5) $$\mathrm{PSSM}=(N_{1},\;N_{2},\ldots ,\;N_{i},\ldots ,\;N_{20})$$

where $N_{i}$ = ${\left(N_{1i},\;N_{2i},\ldots ,\;N_{Li}\right)}^{T}$, (*i* = 1, 2, …, 20). A PSSM contains L × 20 elements, where L denotes the length of an amino acid sequence and 20 columns are owing to 20 amino acids. The $N_{ij}$ of the PSSM element is indicated as a score of jth amino acid in the ith position of the given protein sequence and it can be expressed as $N_{ij}$ = $\sum_{k=1}^{20}p\left(i,k\right)\times q\left(j,k\right)$ where p(i,k) is the appearing frequency value of the $k_{th}$ amino acid at position *i* of the probe, and q(j,k) represents the value of Dayhoff’s mutation matrix [52] between the $j_{th}$ and the $k_{th}$ amino acids. Consequently, the higher the score, the better the conserved position [53,54,55].

In our study, the experiment datasets were built by using PSI-BLAST to transform each protein into a PSSM for detecting PPIs. To obtain more extensive homologous sequences, the e-value parameter of PSI-BLAST was set to 0.001 and chose three iterations. As a result, the PSSM of a protein sequence can be represented as a *M* × 20 matrix, where *M* is the number of residues and each column represents an amino acid [56,57,58,59].

### 3.3. Zernike Moments

Zernike moments have an exciting performance in the field of image recognition for extract image feature, because it is robust against rotation and it can represent information from different angles. In this paper, we first introduced Zernike moments to extract significant information from protein sequences. In this section, Zernike moments and their principal properties are described, and we illustrate how to achieve the rotation invariance. Finally, we describe the process of feature selection.

#### 3.3.1. Invariance of Normalized Zernike Moment

The principle of Zernike moments [60,61,62,63] is Zernike polynomials [64,65,66], that is a set of complete orthogonal polynomials within the unit circle. In two-dimensional space, these polynomials can be expressed as {$V_{nm}\left(x,y\right)$} and expression is as follows:

(6) $$V_{nm}\left(x,y\right)=V_{nm}\left(\rho ,\theta \right)=R_{nm}\left(\rho \right)e^{jm\theta }\;\mathrm{for}\;\rho \leq 1$$

where *n* is a nonnegative integer and *m* is an integer subject to constraints *n*−|*m*| even, |*m*| ≤ *n*. Here, {$R_{nm}\left(\rho \right)$} is a radial polynomial in the form of

(7) $$R_{nm}\left(\rho \right)=\sum_{s=0}^{\left(n-\left|m\right|/2\right)}{\left(-1\right)}^{s}\frac{\left(n-s\right)!}{s!\left(\frac{n+\left|m\right|}{2}-s\right)!\left(\frac{n+\left|m\right|}{2}-s\right)!}\rho^{n-2s}$$

Note that $R_{n,-m}\left(\rho \right)=R_{nm}\left(\rho \right).$The set of polynomials are orthogonal, i.e.,

(8) $$\int_{0}^{2\pi }\int_{0}^{1}V_{nm}^{*}\left(\rho ,\theta \right)V_{pq}\left(\rho ,\theta \right)\rho d\rho d\theta =\frac{\pi }{n+1}\delta_{np}\delta_{mq}$$

With

(9) $$\delta_{ab}=\{\begin{array}{cc} 1 & a=b\\ 0 & otherwise \end{array}$$

The two-dimensional Zernike moments for continuous function *f* (ρ,θ) are the projection of *f* (ρ,θ) onto these orthogonal basis function and denoted by

(10) $$A_{nm}=\frac{n+1}{\pi }\int_{0}^{2\pi }\int_{0}^{1}f\left(\rho ,\theta \right)V_{nm}^{*}\left(\rho ,\theta \right)\rho d\rho d\theta$$

Correspondingly, for a digital function, the two-dimensional Zernike moments are represented by

(11) $$A_{nm}=\frac{n+1}{\pi }\sum_{\left(\rho ,\theta \right)\in unit\;circle}\sum \;f\left(\rho ,\theta \right)V_{nm}^{*}\left(\rho ,\theta \right)$$

To compute the Zernike moments of a PSSM matrix [67,68,69,70], the center of the matrix is taken as the origin and coordinates are mapped into a unit circle, i.e., $x^{2}+y^{2}\leq 1$. Those values of matrix falling outside the unit disk are not used in the computation. Note that $A_{nm}^{*}=A_{n,-m.}$

#### 3.2.2. Introduction of a Zernike Moments Descriptor

When we define $f^{\prime }\left(\rho ,\theta \right)$ as the rotated function, the equivalence between original and rotated function is

(12) $$f^{\prime }\left(\rho ,\theta \right)=\;f\left(\rho ,\theta -\alpha \right)$$

The Zernike moments $A_{nm}^{'}$ of the rotated function $f^{\prime }\left(\rho ,\theta \right)$ become

(13) $$A_{nm}^{'}=A_{nm}e^{-jm\alpha }$$

Equation (13) indicates that Zernike moments only need phase shift on rotation. Therefore, the magnitude of the Zernike moment, |$A_{nm}^{'}$|, can be adopted as rotation-invariant feature.

Therefore, after moving the origin of PSSM matrix into the centroid, we can compute the Zernike moments and the magnitudes of the moments are rotation-invariant [71,72].

#### 3.2.3. Feature Selection

According to the foregoing, we have known that the magnitudes of Zernike moments can be used as rotation-invariant features. One problem that must be considered is how big should *N* be? The lower-order moments extract gross information and high details information are captured by higher-order moments. In our experiments, *N* is set to 12. We can obtain 42 features from each protein sequence. The feature vector $\vec{F}$ be represented as:

(14) $$\vec{F}={\left[\left|A_{11}\right|,\left|A_{22}\right|,\ldots \ldots ,\left|A_{NM}\right|\right]}^{T}$$

where |$A_{nm}$| represent the Zernike moments magnitude. Here, we do not consider the case of *m* = 0, because they do not include useful information regarding the PPIs and Zernike moments with *m* < 0 have not been considered, because they are inferred through $A_{n,-m}=A_{nm}^{*}.$ Hence, the dimension of the feature vector $\vec{F}$ is 42 [73]. The obtained Zernike moments is shown in Table 6.

### 3.4. Related Machine Learning Models

In the field of machine learning, the Support Vector Machines (SVM) [74] are acknowledged as an excellent supervision model in pattern recognition, classification, and regression analysis. However, there are certain apparent disadvantages when using this method: (1) the count of support vectors grows linearly with the scale of the training set; (2) Outputs of the SVMs are not probabilistic; (3) The parameters of kernel function need to be optimized by cross-validation, the procedure wastes a lot of computing resources. Compared with SVM, the Relevance Vector Machines (RVM) [75] based on Bayesian technique can avoid these problems. The RVM method takes advantage of the Bayesian automatic relevance determination (ARD) [76] framework and gives a zero-mean Gaussian prior over every weight $w_{i}$ to produce a sparse solution. However, for a classification problem, the zero-mean Gaussian prior are given over weights for negative and positive classes, which leads to a problem that some training points belonging to negative classes may be given positive weights and vice-versa. Under this circumstance, it may give rise to produce some unreliable vectors for the decision of RVMs. For the sake of addressing this problem and proposing an appropriate probabilistic model for predicting PPIs, we first adopt the Probabilistic Classification Vector Machine (PCVM) classifier which gives different priors over weights for training points that belong to different classes, i.e., the non-negative, left-truncated Gaussian is used for the positive class and the non-positive, right-truncated Gaussian is used for the negative class. PCVM provides many advantages: (1) PCVM produces the probabilistic outputs for each test point; (2) It is effective that PCVM used expectation maximization (EM) algorithm to optimizing kernel parameters; (3) PCVM introduced a sparser model leading to faster performance in the test stage.

### 3.5. PCVM Algorithm

PCVM is a classification model that supervised learning. Hence, we need a set of input-target training pairs ${\left\{x_{i},\;y_{i}\right\}}_{i=1}^{N}$, where $y_{i}$ = {−1, +1} to train a learning model *f* (x; w), which is defined by parameters *W*. The model is a linear combination of *N* basis functions and is represented as

(15) $$f\;(x;\;w)\;=\sum_{i=1}^{N}w_{i}\emptyset_{i,\theta }\left(x\right)+b$$

where the {$\emptyset_{1,\theta }\left(x\right),\ldots \ldots \emptyset_{N,\theta }\left(x\right)$} is basis function, (wherein θ represent the parameter vector of the basis function), the *W* = ${\left(w_{1},\ldots \ldots ,w_{N}\right)}^{T}$ is the parameter of the PCVM model, the *b* is the bias.

In this paper, we adopt the radial basis function (RBF) [77] as the basis and adopt the probit link function $\psi \left(x\right)=\int_{-\infty }^{x}N(t|0,1)dt$ to obtain the binary outputs. Finally, mapping the *f* (x; w) into ψ(x), the expression of the PCVM model becomes:

(16) $$L\;(X;\;w,\;b)=\psi \left(\sum_{i=1}^{N}w_{i}\emptyset_{i,\theta }\left(x\right)+b\right)=\psi \left(\Phi_{\theta }\left(X\right)W+b\right)$$

A truncated Gaussian distribution as a prior is employed over each weight $w_{i}$ as follow

(17) $$p(W|\alpha )=\prod_{i=1}^{N}p\left(w_{i}|\alpha_{i}\right)=\prod_{i=1}^{N}N_{t}(w_{i}|0,\alpha_{i}^{-1})$$

A zero-mean Gaussian distribution as a prior is employed over the bias *b*:

(18) $$p(b|\beta )=N(b|0,\;\beta^{-1})$$

The $N_{t}(w_{i}|0,\alpha_{i}^{-1})$ is a truncated Gaussian function, $\alpha_{i}$ is the precision of the corresponding parameter $w_{i}$, β represents the precision of the normal distribution of *b*. When $y_{i}=+1$, the truncated prior is a non-negative, left-truncated Gaussian, and when $y_{i}=-1$, the prior is a non-positive, right-truncated Gaussian. This can be represented as

(19) $$p(w_{i}|\alpha_{i})=\{\begin{array}{cc} 2N(w_{i}|0,\alpha_{i}^{-1}) & y_{i}w_{i}\geq 0\\ 0 & others \end{array}$$

The gamma distribution is adopted as the hyper prior of *α* and *β*. Using the EM algorithm, assign the parameters of a PCVM model, such as parameters *b*, *W* and *θ*. The EM algorithm is an iterative algorithm, which is used to estimate the maximum likelihood or maximum posterior probability involving latent variables. For more details about the PCVM theory, please refer to [78,79].

### 3.6. Initial Parameter Selection and Training

The PCVM algorithm has only one parameter, *θ*, which can be optimized automatically in the training process. However, the EM algorithm is susceptible to initial point and trap in local maxima. Choosing the best initialization point is an effective method to avoid the local maxima. We train a PCVM model with eight initialization points over the five training folds of each data. Hence, we obtain a 5 × 8 matrix of parameters, where the rows represent the folds and the columns represent the initializations. For each row, we select the results of the lowest test error. Hence, we find only five points, and then, we select the medium over those parameters. We have experimental obtained the optimal initial value θ which is seted as 3.6 on the *Yeast* dataset and 1.18 on the *H. pylori* dataset.

## 4. Conclusions

Considering time, efficiency and economy, the use of computational methods based on protein amino acid sequences to predict PPIs has attracted the attention of researchers. The computational method is playing an important role in proteomics research, because it saves manpower and material resources and is more accurate and efficient. In this paper, we introduce an accurate computational method based on protein sequence. It is established by using a PCVM classifier combined with a Zernike moments descriptor on the PSSM. The experiments showed that the performance of our proposed method achieves a high classification accuracy and is superior to the SVM. The main improvements of the developed approach come from adopting a Zernike moments descriptor as feature extraction approach that can capture multi-angle useful and representative information. More than this, the use of a PCVM classifier ensures more reliable and accurate recognition, because the use of the truncated Gaussian priors can lead to obtaining robust and sparse results—the number of support vectors is less than SVM, and the probabilistic outputs produced by PCVM can assess the uncertainty of prediction on the skewed dataset. In addition, the parameter optimization procedure of the PCVM not only can improve the performance, but also save effort to do cross-validation. Due to the outstanding performance of the Zernike moments descriptor and PCVM, our method can improve the PPIs accuracy rate. All in all, our proposed method is highly efficient and stable and can be a useful tool for predicting PPIs.

## Figures and Tables

**Table 1:** Fivefold cross validation results using the proposed method on *Yeast* dataset.

Testing Set	Acc (%)	Sen (%)	Pre (%)	MCC (%)
1	96.38	97.21	95.57	93.02
2	94.05	95.23	92.77	88.81
3	93.07	96.73	90.27	87.06
4	94.46	94.20	94.71	89.53
5	94.42	92.26	96.26	89.46
Average	94.48 ± 1.2	95.13 ± 2.0	93.92 ± 2.4	89.58 ± 2.2

**Table 2:** Fivefold cross validation results using the proposed method on *H. Pylori* dataset.

Testing Set	Acc (%)	Sen (%)	Pre (%)	MCC (%)
1	89.54	92.11	86.82	81.24
2	92.11	92.68	91.41	85.46
3	91.08	91.16	91.16	83.75
4	91.42	92.25	90.34	84.31
5	92.12	92.04	93.23	85.42
Average	91.25 ± 1.1	92.05 ± 0.6	90.06 ± 2.4	84.04 ± 1.7

**Table 3:** Five-fold cross-validation results by using two models on the *Yeast* dataset.

Model	Testing Set	Acc (%)	Sen (%)	Pre (%)	MCC (%)
Probabilistic Classification Vector Machines (PCVM)	1	96.38	97.21	95.57	93.02
	2	94.05	95.23	92.77	88.81
	3	93.07	96.73	90.27	87.06
	4	94.46	94.20	94.71	89.53
	5	94.42	92.26	96.26	89.46
	Average	94.48 ± 1.2	95.13 ± 2.0	93.92 ± 2.4	89.58 ± 2.2
Support Vector Machin (SVM)	1	89.23	87.75	90.27	80.76
	2	90.48	88.73	91.49	82.74
	3	87.62	87.37	88.07	78.30
	4	89.63	88.05	90.97	81.40
	5	89.60	85.79	93.23	81.32
	Average	89.31 ± 1.7	87.54 ± 1.1	90.81 ± 1.9	80.91 ± 1.62

**Table 4:** Practical predicting results of different methods on the *Yeast* dataset.

Model	Testing Set	Acc (%)	Sen (%)	Pre (%)	MCC (%)
Guo [20]	Auto Covariance (ACC)	89.33 ± 2.67	89.93 ± 3.68	88.87 ± 6.16	N/A
	auto covariance (AC)	87.36 ± 1.38	87.30 ± 4.68	87.82 ± 4.33	N/A
Yang [23]	Cod1	75.08 ± 1.13	75.81 ± 1.20	74.75 ± 1.23	N/A
	Cod2	80.04 ± 1.06	76.77 ± 0.69	82.17 ± 1.35	N/A
	Cod3	80.41 ± 0.47	78.14 ± 0.90	81.66 ± 0.99	N/A
	Cod4	86.15 ± 1.17	81.03 ± 1.74	90.24 ± 1.34	N/A
You [24]	Principal Component Analysis-Ensemble Extreme Learning Machines (PCA-EELM)	87.00 ± 0.29	86.15 ± 0.43	87.59 ± 0.32	77.36 ± 0.44
Wong [30]	Rotation Forest (RF) + Property Response-Local Phase Quantization (PR-LPQ)	93.92 ± 0.36	91.10 ± 0.31	96.45 ± 0.45	88.56 ± 0.63
Proposed Method	PCVM	94.48 ± 1.20	95.13 ± 2.00	93.92 ± 2.40	89.58 ± 2.20

**Table 5:** Practical predicting results of different methods on the *H. Pylori* dataset.

Model	Acc (%)	Sen (%)	Pre (%)	MCC (%)
Nanni [23]	83.00	86.00	85.10	N/A
Nanni [32]	84.00	86.00	84.00	N/A
Nanni and Lumini [25]	86.60	86.70	85.00	N/A
Z-H You [29]	87.50	88.95	86.15	78.13
L Nanni [24]	84.00	84.00	84.00	N/A
Proposed Method	91.25	92.05	90.06	84.04

**Table 6:** List of Zernike Moments (ZMs) sorted by *n* and *m* in sequence for the case where (*n*, *m*) = (12, 12).

*N*	Moments	No.	*N*	Moments	No.
1	$A_{11}$	1	7	$A_{71},A_{73},A_{75},A_{77}$	4
2	$A_{22}$	1	8	$A_{82},A_{84},A_{86},A_{88}$	4
3	$A_{31},A_{33}$	2	9	$A_{91},A_{93},A_{95},A_{97},A_{99}$	5
4	$A_{42},A_{44}$	2	10	$A_{10,2},A_{10,4},A_{10,6},A_{10,8},A_{10,10}$	5
5	$A_{51},A_{53},A_{55}$	3	11	$A_{11,1},A_{11,3},A_{11,5},A_{11,7},A_{11,9},A_{11,11}$	6
6	$A_{62},A_{64},A_{66}$	3	12	$A_{12,2},A_{12,4},A_{12,6},A_{12,8},A_{12,10},A_{12,12}$	6
